# Semiflex-assisted vacuum therapy for perianal fistulas: the Semiflex pilot study

**DOI:** 10.1007/s10151-025-03240-1

**Published:** 2025-12-08

**Authors:** A. J. M. Pronk, J. Y. van Oostendorp, I. J. M. Han-Geurts, S. Madelska, C. J. Buskens, W. A. Bemelman

**Affiliations:** 1 Department of Surgery, Amsterdam University Medical Center, Location VUMC, De Boelelaan 1117, 1081HV Amsterdam, The Netherlands; 2 Department of Surgery, Proctos Kliniek, Bilthoven, The Netherlands; 3 Semiflex Dome System Ltd, Poznań, Poland

**Keywords:** Perianal fistulas, Vaccuumtherapy, Surgery

## Abstract

**Introduction:**

Perianal fistulas often require multiple surgical interventions because of their chronic nature. Various sphincter-sparing techniques achieve clinical closure rates of up to 70%, yet recurrence remains a major challenge. Vacuum-assisted closure (VAC) therapy has shown promise in wound healing, but its application in perianal fistulas remains largely unexplored. The Semiflex catheter was developed to facilitate outpatient vacuum therapy without the need for general anesthesia during catheter exchanges. This pilot study aimed to evaluate the feasibility and clinical applicability of the Semiflex catheter in perianal fistula management.

**Methods:**

The Semiflex pilot study was a two-part feasibility trial. The first part assessed proof of principle in ten patients, while the second part, a multicentre study, aimed to confirm feasibility in 20 patients. Feasibility included smoothness of insertion and changing of the Semiflex catheters, capability of proper fixation of the Semiflex catheter, maintaining vacuum for more than 48 h, and compliance to the therapy in terms of pain and discomfort. The protocol was scored feasible if at least 50% of the exchanges met all these criteria in at least 70% of patients. Secondary outcomes included clinical fistula closure, radiological healing, and treatment-related adverse events.

**Results:**

Twenty patients were included (median age 39.5 years; 70% Crohn’s disease). Thirteen Semiflex treatments were scored as feasible, below the predefined threshold. Clinical fistula closure was observed in 50% of patients, but none showed radiological healing at 3 months. One serious adverse event occurred, requiring early treatment discontinuation, while minor complications, including local skin reactions and pressure sores, were managed conservatively.

**Conclusions:**

Semiflex therapy was feasible in a subset of patients and allowed outpatient treatment. However, maintaining vacuum and achieving long-term fistula closure remains challenging. While Semiflex may have a role in perianal fistula management, further research is needed to refine patient selection and optimize its application.

## Introduction

Perianal fistulas typically arise from cryptoglandular sepsis or as a complication of Crohn’s disease (CD). Pain and discharge of pus, stool or blood are common symptoms which severely reduce the quality of life in these patients [1, 2]. Permanent closure of perianal fistulas often requires multiple surgical interventions. Several sphincter-sparing procedures are available, of which ligation of the intersphincteric fistula tract (LIFT) and the mucosal advancement flap (AF) are the most commonly used techniques. Clinical closure rates up to 70% have been reported for both cryptoglandular and CD-related fistulas. While these outcomes may seem quite satisfactory, recurrence remains a considerable challenge, affecting up to a quarter of these patients [3]. In an effort to improve treatment success, mesenchymal stem cell therapy was introduced as a promising new treatment approach for CD-related perianal fistulas. However, after an initial randomized controlled trial with significantly increased combined closure rates when compared to placebo treatment, a subsequent study failed to demonstrate an additional advantage of this expensive treatment and recently darvadstrocel was withdrawn from the European market [4, 5]. Thus, there is a pressing need for novel therapeutic strategies that can achieve permanent fistula closure.

**Fig. 1:**
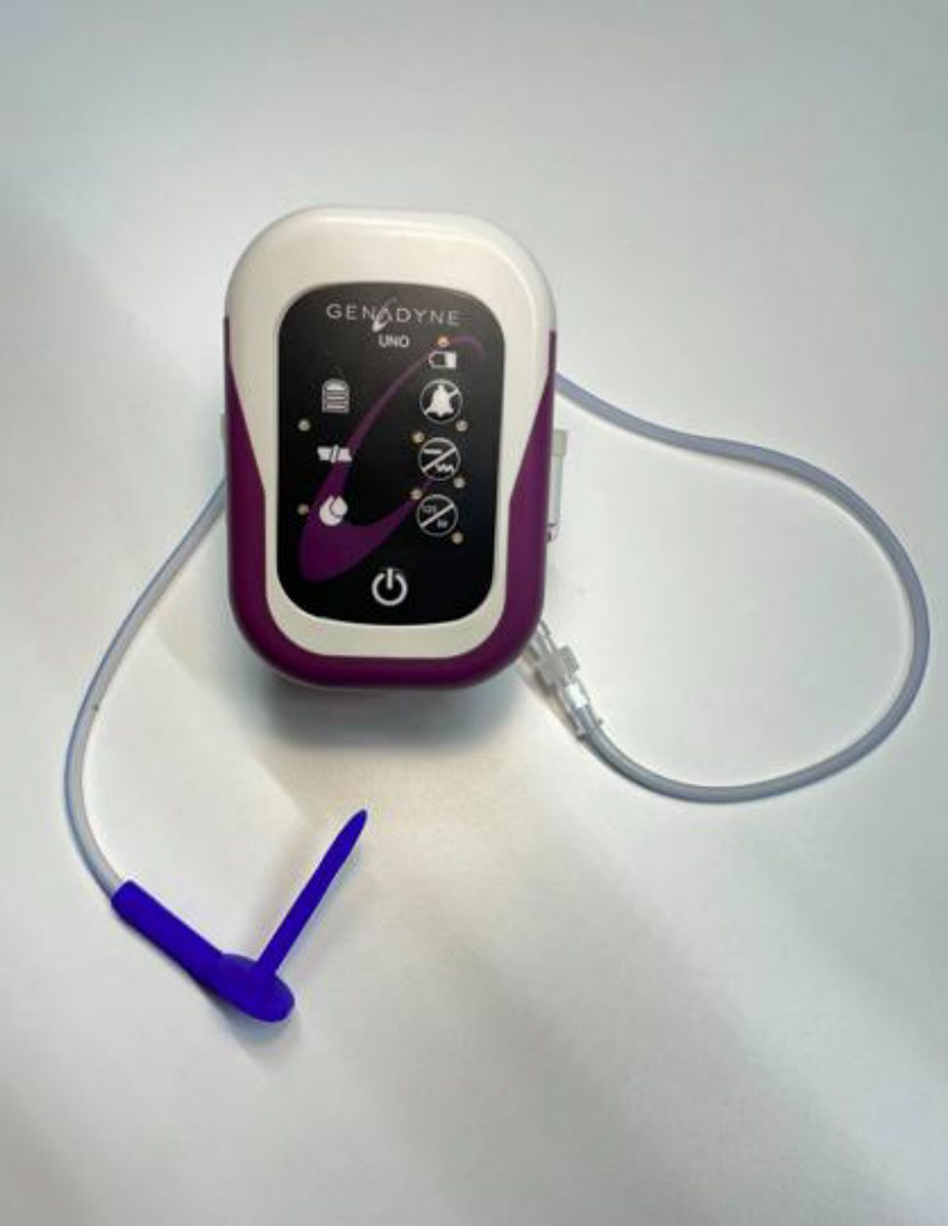
The Semiflex catheter system

Vacuum-assisted closure (VAC) therapy has become one of the main pillars for management of a wide variety of wound healing problems [6]. VAC promotes wound healing through four primary mechanisms including macro- and microdeformation of wound tissue, fluid removal and stabilization of the wound environment [7]. These mechanisms, along with their secondary effects, such as reduced bacterial load, granulation tissue formation and angiogenesis, promote wound healing. To date, only one study has investigated the use of VAC therapy for perianal fistulas. This cohort study included 24 patients and found a clinical closure rate of 75% using homemade vacuum catheters [8]. The downside of these catheters was that each catheter exchange required general anaesthesia. To address this limitation, the Semiflex catheter was developed, allowing vacuum therapy to be administered in an outpatient setting, as catheter exchanges can be performed without anaesthesia. The aim of this study was to determine the feasibility and clinical applicability of this newly developed Semiflex catheter (Fig. 1).

## Methods

### Study design

The Semiflex pilot study was a feasibility study to test a novel catheter set for vacuum therapy of perianal fistulas. The trial consisted of two parts. In the first part of the study, ten patients were included at the Amsterdam UMC to investigate if the catheter met the proof of principle. If the proof of principle was met, the second part of the trial continued as a multicentre study to confirm the external validity of the vacuum application in one other centre, the Proctos clinics, a specialized proctology centre (Bilthoven, the Netherlands). The study was conducted in accordance with Good Clinical Practice guidelines and with the principles of the Declaration of Helsinki. The Medical Ethic committee of the Amsterdam UMC approved the study for both the Amsterdam UMC and the Proctos clinics. The study protocol is available online (NCT06446635).

### Study population

Eligible patients were aged ≥ 18 and < 80 years and had a perianal fistula. Perianal fistulas either idiopathic/cryptoglandular or related to CD were eligible for inclusion. Exclusion criteria included the presence of more than two external perianal fistula openings, rectovaginal fistulas, a life expectancy of less than 2 years, or cognitive impairments (such as dementia) that would hinder the ability to understand the given information or provide informed consent. All participants provided written informed consent prior to enrolment.

### Surgical intervention and follow-up

**Fig. 2:**
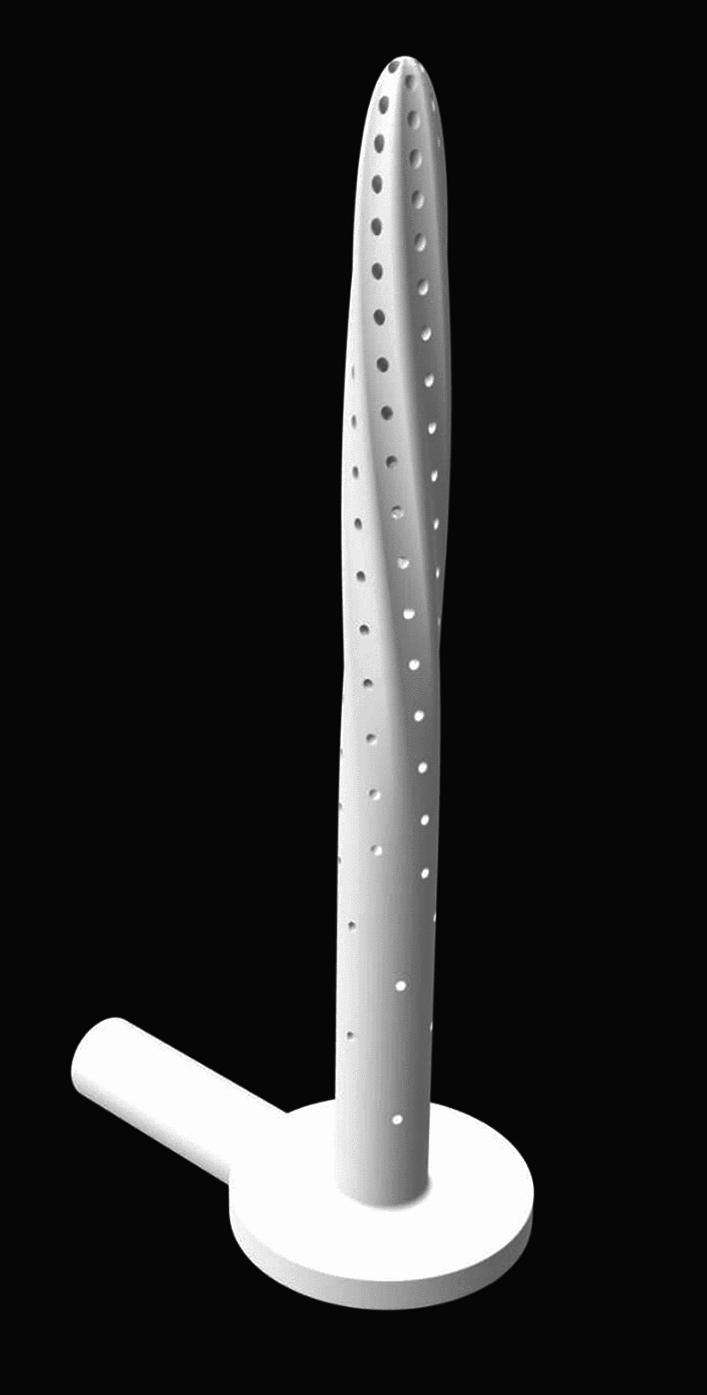
Detailed Semiflex catheter 3D visualization

Placement of the Semiflex catheter was performed under general or spinal anaesthesia in the operating theatre. The procedure started with thorough curettage of the fistula tract, followed by de-epithelialization and closure of the internal opening using Vicryl 2–0 sutures. The length of the tract was then measured to select an appropriately sized Semiflex catheter. The Semiflex catheter was inserted with its plate fixed on a Renasys Adhesive gel patch (Smith & Nephew) and connected to a vacuum pump set to a vacuum pressure of 125 cmH_2_O using an IV tube. Afterwards, a transparent film dressing was placed over the Semiflex catheter and the first part of the tube to secure the vacuum seal. In total, 30 Semiflex catheter sizes are available, ranging in length from 0 to 87 mm (Fig. 2). Each catheter is 3 mm larger and 0.15 mm bigger in diameter compared to the previous one.

Every other day, the Semiflex catheter was exchanged with a shorter and smaller version. These exchanges were performed either in the outpatient setting or at home by a spouse. The exchanges continued until the entire fistula tract was treated. The duration of the therapy depended on the length and width of the fistula tract. One month after completing the Semiflex treatment, patients attended an outpatient visit to determine clinical closure of the fistula. A follow-up magnetic resonance imaging (MRI) was scheduled 3 to 6 months later to assess radiological healing of the fistula.

### Patient demographics and outcome variables

Data on patient demographics, medication use, prior perianal interventions, and details of the Semiflex treatment and follow-up were collected from medical records. Patient demographics included sex, age, smoking history, duration of CD, CD location according to the Montreal classification, and fistula characteristics. The two parts of the Semiflex pilot study had different primary outcomes. The primary outcome of the first part was to determine whether the Semiflex catheter meets the proof of principle with respect to smoothness of insertion and exchanging of the Semiflex catheter, capability of proper fixation of the Semiflex catheter, maintaining vacuum for more than 48 h, and compliance to the therapy in terms of pain and discomfort. During each exchange it was assessed whether the Semiflex catheter was still correctly inserted and maintained vacuum pressure. The medical practitioner or spouse determined if the catheter could be smoothly inserted and properly secured, while the patient rated the pain during the exchange on a scale from 0 (no pain) to 10 (severe pain). A pain score ≤ 5 was considered successful. Proof of principle was achieved if at least 50% of the exchanges met these criteria in at least five of ten patients.

The second part of the study focused on the feasibility of the Semiflex catheter. Feasibility was confirmed if at least 50% of exchanges met the same success criteria in at least 14 of 20 patients.

Secondary outcomes were clinical fistula closure, defined as closure of the external opening without discharge of pus or faeces on palpation, and radiological healing, defined as complete fibrosis of the fistula tract on MRI, complications, the ability to train the spouse to perform catheter exchanges independently, and a pain/uncomfortability score at 3 months post Semiflex treatment.

### Statistical methods

Since this was a pilot study, no formal sample size calculation was performed. All data were collected using the electronic data management system Castor EDC (https://www.castoredc.com). Patient characteristics and outcome parameters were summarized using descriptive statistics. Non-parametric data were presented as median and interquartile range (IQR), while categorical data were expressed as frequencies and percentages. Patient and disease characteristics possibly associated with a feasible treatment were analysed by univariate analyses using chi square for categorical data and the Mann–Whitney* U* test for non-parametric data. All statistical analyses were performed using the Statistical Package for the Social Sciences (SPSS, version 28.0.1). If the proof of principle was confirmed in the first part of the study, data from both parts were combined for analysis.

## Results

### Baseline characteristics

In total, 20 patients were included, of whom seven (35.0%) were male. The median age at time of surgery was 39.5 years (IQR 29.0–53.5), and none of the patients were smokers. The median duration of perianal fistula symptoms was 5.5 years (IQR 2.0–14.5), with a median of 4.0 prior perianal interventions (IQR 1.0–9.0). Additionally, four patients (20.0%) had an ostomy at the time of surgery. The majority of patients (70.0%) suffered from CD at the time of surgery, with a median disease duration of 9.0 years (IQR 5.0–14.8). Fifteen patients (75.0%) were using medication at time of surgery, with most of them using anti-tumour necrosis factor (TNF; 60.0%, 9/15 patients). Among patients with CD, 42.9% (6/14 patients) were diagnosed with L3 disease. Most patients (60.0%) had a seton in place at the time of surgery, with a median duration of 12.0 months (IQR 4.0–20.8). No complications occurred during surgery. The median duration of Semiflex catheter therapy was 25.0 days (IQR 12.5–35.0), with a median of 13.0 Semiflex exchanges (IQR 5.8–15.0). Nine patients (45.0%) were treated at home, with catheter exchanges performed by a spouse (Table 1).

**Table 1:** Baseline characteristics

Baseline characteristics	*N* = 20
Male sex, *n* (%)	7 (35.0%)
Age, years^a^	39.5 (29.0–53.5)
Smoking at moment of surgery, *n* (%)	0 (0%)
History of Crohn’s disease, *n* (%)	14 (70.0%)
Duration Crohn’s disease, years^a^	9.0 (5.0–14.8)
Location of Crohn’s disease, *n* (%)	
L1 Ileum	4 (28.6%)
L2 Colon	3 (21.4%)
L3 Ileum and colon	6 (42.9%)
Missing	1 (7.1%)
Use of medication, *n* (%)	
Antibiotics	1 (5.0%)
Anti-TNF treatment	9 (45.0%)
Immunomodulator	4 (20.0%)
Combination of anti-TNF treatment and immunomodulator	1 (5.0%)
None	5 (25.0%)
Duration of symptoms, years^a^	5.5 (2.0–14.5)
Number of prior perianal surgeries^a^	4.0 (1.0–9.0)
Seton at the time of surgery, *n* (%)	12 (60.0%)
Seton drainage duration, months^a^	12.0 (4.0–20.8)
Ostomy at time of surgery, *n* (%)	4 (20.0%)
Type of fistula tract, *n* (%)	
Transsphincteric	19 (95.0%)
Intersphincteric	1 (5.0%)
Therapy length, days^a^	25.0 (12.5–35.0)
Number of catheter exchanges^a^	13.0 (5.8–15.0)
Treatment at home^a^	9 (45.0%)

*n*   number of patients

^a^Values are presented median (interquartile range)

### Feasibility

In all patients (100%, 20/20 patients), the catheter could be inserted smoothly. In 95% of patients (19/20 patients), proper fixation of the catheter was achieved after all exchanges. The patient in whom fixation was not possible had undergone 12 prior perianal interventions, resulting in extensive scaring of the perianal region. In 14 patients (70%), the Semiflex catheter maintained vacuum in more than 50% of the exchanges. The mean VAS score across all exchanges was 1.6 (± SD 1.3). In 19 patients (95%), all exchanges were scored with a pain score ≤ 5. Overall, 13/20 treatments were scored as feasible (Table 2). The median pain/uncomfortability score 3 months after Semiflex treatment was 2.5 (IQR 0–4.5), based on 16/20 (80%) response rate.

**Table 2:** Feasibility scored per patient

Patient nr.	Smoothly insert the catheter	Properly fixate the catheter	Maintained vacuum for > 48 h	Pain score ≤ 5	Feasible
1	Yes	100%	100%	100%	Yes
2	Yes	100%	89%	100%	Yes
3	Yes	100%	85%	100%	Yes
4	Yes	0%	100%	100%	No
5	Yes	100%	100%	100%	Yes
6	Yes	100%	87%	100%	Yes
7	Yes	100%	64%	100%	Yes
8	Yes	100%	8%	100%	No
9	Yes	100%	40%	100%	No
10	Yes	100%	0%	100%	No
11	Yes	100%	100%	100%	Yes
12	Yes	100%	0%	100%	No
13	Yes	100%	100%	100%	Yes
14	Yes	100%	100%	84%	Yes
15	Yes	100%	88%	100%	Yes
16	Yes	100%	53%	100%	Yes
17	Yes	100%	60%	100%	Yes
18	Yes	100%	0%	100%	No
19	Yes	100%	0%	100%	No
20	Yes	100%	50%	100%	Yes

Univariate analyses were performed for various variables as presented in Table 3. Although not statistically significant, the percentage of patients with an ostomy at time of surgery was higher in patients in whom the Semiflex treatment was scored as not feasible (28.9% vs. 15.4%). In addition, patients for whom Semiflex treatment was scored as not feasible had a higher median number of prior perianal surgeries (7.0; IQR 2.5–8.3) compared to those for whom it was scored as feasible (3.0; IQR 1.0–9.0). The presence of a seton during surgery and the location of Semiflex exchange (at home or outpatient clinic) did not appear to influence feasibility.

**Table 3:** Univariate analyses for feasibility

	Feasible (*n* = 13)	Not feasible (*n* = 7)	*p* value
Crohn’s disease, *n* (%)	9 (69.2)	5 (71.4)	0.664
Number of prior perianal surgeries^a^	3.0 (1.0–9.0)	7.0 (2.5–8.3)	0.579
Ostomy at time of surgery, *n* (%)	2 (15.4)	2 (28.9)	0.439
Seton at the time of surgery, *n* (%)	8 (61.5)	4 (57.1)	0.608
Treatment at home^a^, *n* (%)	6 (46.1)	3 (42.9)	0.630

*n*  number of patients

^a^Values are presented median (interquartile range)

### Perianal fistula closure

Following Semiflex treatment, ten patients (50%) achieved clinical closure of the fistula after a median of 1.5 months (IQR 1.0–3.8). All patients with clinically closed fistulas underwent an MRI; however, none (0%) showed radiological healing of the fistula.

### (Serious) adverse events and reinterventions

During treatment, one serious adverse event occurred: suture failure at the level of the internal opening in one patient, which required a reintervention for replacement 5 days after the index surgery. However, during the procedure, it was determined that the suture could not be replaced because of excessive tension on the wound. As a result, Semiflex treatment was discontinued for this patient and a loose seton was placed instead. Additionally, two other patients required a reintervention within 3 months as a result of failure of perianal fistula closure: one underwent mesenchymal stem cell treatment, and the other underwent fistula laser-assisted closure (FiLaC).

In total, ten adverse events were reported during treatment and follow-up. These included a small abscess near the internal opening, which was drained in the outpatient clinic; one patient experienced postoperative obstipation which was managed conservatively; in a second suture failure at the level of the internal opening occurred, leading to discontinuation of Semiflex treatment; two patients developed pressure sores near the external fistula opening; two patients had allergic or extensive skin reactions to the transparent film; and three patients who reported mild skin redness. The pressure sores and skin reactions could all be treated with Duoderm under the transparent film to protect the skin.

## Discussion

This pilot study evaluated the feasibility and applicability of the Semiflex catheter, which enables vacuum therapy for perianal fistulas. A total of 20 patients were included, of whom 13 (65%) were considered to have undergone feasible treatment. Following Semiflex catheter treatment, 10 patients (50%) had a clinically closed fistula after a median of 1.5 months. However, none of the patients with clinical closure showed radiological healing of the fistula. One serious adverse event occurred during treatment, requiring reintervention.

The Semiflex treatment did not meet the predefined feasibility threshold of 14 out of 20 patients, as was established in the original study design. This may be attributed to the high median number of previous perianal surgeries of 4.0 (IQR 1.0–9.0) and the fact that four patients had an ostomy at the time of surgery, indicating severe disease. Patients in whom the treatment was considered not feasible had a higher proportion of ostomies and a larger median number of prior perianal surgeries. The extensive perianal scarring from multiple previous surgeries hinders the fixation of the Semiflex catheter and/or the creation of a vacuum, thereby reducing the feasibility rate.

A key challenge with the Semiflex catheter was maintaining vacuum for more than 48 h. As previously mentioned, extensive scarring of the perianal area compromises the airtight seal of the fixation of the Semiflex. Additionally, when the external fistula opening is located close to the anus, there may be insufficient space to properly secure the catheter. To improve outcomes, careful assessment of the external fistula opening’s location and the perianal area’s condition should be conducted prior to initiating Semiflex treatment and guide patient selection. Another factor contributing to the loss of vacuum was the detachment of the transparent film used to seal the Semiflex catheter. This detachment could result from patient movement, cleaning of the perianal area (i.e. after defecation), or (allergic) skin reactions to the film. For future applications, the use of a more adhesive, hypoallergenic film may help reduce the risk of detachment and maintain the vacuum more effectively.

To date, only one other study has evaluated vacuum therapy for perianal fistulas [8]. This cohort study included 24 patients and reported a clinical closure rate of 75% using homemade vacuum catheters, which is higher than the 50% observed in this study. This discrepancy may be explained by differences in patient populations; only 37.5% of patients in the other study had undergone prior perianal surgery, so 62.5% were treated for a primary perianal fistula, representing a less therapy-refractory cohort. In contrast, the 50% closure rate observed with Semiflex treatment is notable given the challenging nature of this cohort. All included patients had failed multiple attempts of surgical closure, some of which also included stem cell therapies.

It is important to note that none of the patients in this study achieved radiological healing on MRI, the ultimate treatment goal for perianal fistulas associated with CD owing to its strong predictive value for long-term clinical closure [9, 10]. The MRIs in this pilot study were performed 3 months after treatment with the Semiflex catheter. Previous studies have suggested that longer follow-up periods may yield higher rates of radiological healing [11, 12]. Therefore, the timing of our MRIs at 3 months may have been premature, and future studies should include imaging at later intervals, to better capture potential improvements in radiological outcomes.

Although the Semiflex treatment did not meet the predefined feasibility criteria, it may still hold potential as part of a sphincter and tissue-sparing treatment strategy owing to its distinctive attributes. Minimal invasive drainage of the external fistula tract avoids troublesome and long-term woundcare typically associated with fistulectomy. Its ability to avoid sphincter damage aligns with the goals of preserving continence in patients with complex fistulas. Moreover, the treatment is relatively easy to learn and perform, making it feasible for application in a home setting with proper training. The healing rates suggest that it may not be suitable as a standalone closure procedure for therapy-refractory fistulas, nor as a first-line option for primary disease, given the healing rates of established sphincter-sparing techniques (LIFT, AF).

Nevertheless, the Semiflex catheter could play a valuable role in the broader management of complex cases. For instance, it may be particularly beneficial in treating fistulas with wide-diameter tracts or supralevator extensions as a supportive (or pre-definitive) treatment option. It could also serve as a drainage technique following procedures such as LIFT or AF, avoiding fistulectomy of the external tract, simplifying wound care by eliminating the need for frequent rinsing. Finally, it could prove effective in draining perianal abscesses while reducing the need for extensive incisions and minimizing healing complications.

The strength of the Semiflex catheter system is that catheters can be exchanged without the need of general anaesthesia, with a mean VAS of all exchanges of 1.6. After brief training, exchanges can be executed by spouses or home care nurses. Catheter fixation and maintaining vacuum has to be improved in order to be able to include patients with less favourable locations of the external opening and a scarred perineum.

A few limitations of the current study must be acknowledged. First, the small sample size of 20 patients and the short follow-up period of 3 months limit the generalizability of our findings and can yield statistically insignificant results. Second, as a new technique, the Semiflex treatment comes with a learning curve, as with any novel surgical approach. Proficiency in catheter placement, careful patient selection for tailored treatment, and training of caregivers or spouses are essential. Practical guidance on maintaining vacuum functionality, such as using adhesive strips to quickly re-establish vacuum or following careful cleaning routines, will also be essential for optimizing outcomes. Addressing these aspects through structured training and standardized protocols could help reduce some of the initial challenges associated with introducing this new technique.

In conclusion, the Semiflex treatment was deemed feasible in 65% of patients, indicating that the treatment protocol, as currently defined, did not meet our predefined feasibility criteria. Nevertheless, it is the only existing vacuum system that can be inserted and exchanged in fistulising disease without the need of anaesthesia or a natural orifice e.g. Endosponge treatment of anastomotic fistula [13]. While 50% of patients had a clinically closed fistula, none achieved radiological healing at 3 months follow-up. The treatment is safe and relatively straightforward to perform, even in a home setting. Although the Semiflex catheter may have potential in future treatment strategies, its precise role within the therapeutic armamentarium remains to be determined. It could potentially play a role in draining the external tract, thus avoiding fistulectomy after closure methods like primary closure of the internal opening, AF and LIFT. Further research is needed to determine its optimal application and to explore other potential uses.

## Data Availability

No datasets were generated or analysed during the current study.
